# Baicalin Depresses the Sympathoexcitatory Reflex Induced by Myocardial Ischemia via the Dorsal Root Ganglia

**DOI:** 10.3389/fphys.2018.00928

**Published:** 2018-07-17

**Authors:** Lifang Zou, Xinyao Han, Shuangmei Liu, Yingxin Gong, Bing Wu, Zhihua Yi, Hui Liu, Shanhong Zhao, Tianyu Jia, Lin Li, Huilong Yuan, Liran Shi, Chunping Zhang, Yun Gao, Guilin Li, Hong Xu, Shangdong Liang

**Affiliations:** ^1^Department of Physiology, Medical School of Nanchang University, Nanchang, China; ^2^Jiangxi Provincial Key Laboratory of Autonomic Nervous Function and Disease, Nanchang, China; ^3^First Clinical Department, Medical School of Nanchang University, Nanchang, China; ^4^Department of Cell Biology, Medical School of Nanchang University, Nanchang, China

**Keywords:** P2Y_12_ receptor, dorsal root ganglia, satellite glial cells, myocardial ischemia, sympathoexcitatory reflex

## Abstract

Myocardial ischemia (MI) is one of the major causes of death in cardiac diseases. Purinergic signaling is involved in bidirectional neuronal-glial communication in the primary sensory ganglia. The sensory neuritis of cardiac afferent neurons in cervical dorsal root ganglion (cDRG) interacts with cardiac sympathetic efferent postganglionic neurons, forming feedback loops. The P2Y_12_ receptor is expressed in satellite glial cells (SGCs) of DRG. Baicalin is a major active ingredient extracted from natural herbal medicines, which has anti-inflammatory and strong anti-oxidation properties. In this study we investigated the effect of baicalin on P2Y_12_ receptor in the cervical DRG SGC-mediated sympathoexcitatory reflex, which is increased during MI. The results showed that the expression of P2Y_12_ receptor mRNA and protein in DRG, and the co-localization values of P2Y_12_ receptor and glial fibrillary acidic protein (GFAP) in cDRG SGCs were increased after MI. The activated SGCs increased IL-1β protein expression and elevated Akt phosphorylation in cDRG. Baicalin treatment inhibited the upregulation of the P2Y_12_ receptor, GFAP protein and Akt phosphorylation in cDRG neurons/SGCs. The stellate ganglia (SG) affect cardiac sympathetic activity. Baicalin treatment also decreased the upregulation of the P2Y_12_ receptor, GFAP protein in the SG. The P2Y_12_ agonist, 2Me-SADP, increased [Ca^2+^]_i_ in HEK293 cells transfected with the P2Y_12_ receptor plasmid and SGCs in cDRG. These results indicate that application of baicalin alleviates pathologic sympathetic activity induced by MI via inhibition of afferents in the cDRG.

## Introduction

Myocardial ischemia (MI) is one of the major causes of death in cardiac diseases (Hausenloy and Yellon, 2013). ATP is released during an MI and generates its physiological actions through the activation of ionotropic purinergic P2X and metabotropic P2Y receptors (Burnstock, 2007). The sensory neurites of cardiac neurons are located throughout both ventricles and transfer their information via dorsal root ganglia (DRG) to the spinal cord (Huang et al., 1996; Pather et al., 2003). Experimental coronary occlusion elicits sympathoexcitatory reflexes via the activation of sensory endings in the ischemic left ventricle, which has afferent fibers traveling to the spinal cord via primary afferents (Armour, 1999; Pan and Chen, 2002; Hoover et al., 2008). We reported previously that P2X_3_ and P2X_7_ receptors, activated by ATP in stellate ganglia (SG) neurons and cervical DRG neurons, participated in signal transmission resulting from MI damage (Li et al., 2011; Liu et al., 2013, 2014; Tu et al., 2013).

Satellite glial cells (SGCs) are the most abundant cell type in DRG (Costa and Moreira, 2015). SGCs can receive signals from other cells and respond to changes in their environment, and activation of SGCs will also influence neighboring neurons (Hanani, 2005). Following peripheral nerve injury, glial fibrillary acidic proteins (GFAPs) are augmented, which is a marker for SGC activation (Hanani, 2005; Jasmin et al., 2010). Purinergic signaling is involved in bidirectional neuronal-glial communication in primary sensory ganglia (Fields and Burnstock, 2006; Verderio and Matteoli, 2011; Rajasekhar et al., 2015; Shi et al., 2017). Upregulation of P2X_7_ receptors in SGCs mediates the sympathoexcitatory reflex via sensory-sympathetic coupling between cervical DRG nerves and nerves of the cervical sympathetic ganglia (Kong et al., 2013; Liu et al., 2013; Tu et al., 2013). P2Y_12_ receptors are expressed in SGCs of DRG (Kobayashi et al., 2008; Katagiri et al., 2012). Additionally, P2Y_12_ receptors expressed in SCG are involved in mediating the sympathoexcitatory reflex (Zou et al., 2017). DRGs have primary sensory neurons, however, the role of the P2Y_12_ receptor in DRG SGCs during MI remains to be investigated.

Baicalin is a major active ingredient of the natural herbal medicine *Scutellaria baicalensis* Georgi, which has anti-bacterial, anti-inflammatory and strong anti-oxidative properties (Krakauer et al., 2001). Baicalin has been investigated in various pathological conditions (Gao et al., 1999, 2001; Srinivas, 2010). Studies showed that baicalin pretreatment reduces the damage to the liver and brain caused by ischemia-reperfusion (Kim et al., 2010; Xue et al., 2010; Cao et al., 2011). Baicalin pretreatment mitigates cardiomyocyte apoptosis in cultured neonatal rat cells via an anti-inflammatory mechanism and it has also been reported to reduce hypoxia-reoxygenation-induced cardiomyocyte apoptosis (Lin et al., 2010). We also reported that baicalin had cardioprotective properties (Zhang et al., 2015). Here we investigate the effects of P2Y_12_ receptors in DRG satellite glial cells during the MI-induced sympathoexcitatory reflex and the role of baicalin therein.

## Materials and Methods

### Animals and Myocardial Ischemia Models

Sprague-Dawley (SD) rats (180–220 g) were used in all experiments. The use of animals was reviewed and approved by the Animal Care and Use Committee of the Medical College of Nanchang University. Experiments were performed in accordance with the guidelines of the United States National Institutes of Health (NIH) regarding the care and use of animals for experimental procedures. The rats were randomly divided into five groups (6–8 rats per group): a control plus baicalin group (Control + baicalin); a sham group (Sham); a myocardial ischemic group (MI); a group of myocardial ischemic rats treated with vehicle normal saline (NS) (MI + NS); and a group of myocardial ischemic rats treated with baicalin (MI + baicalin). MI was induced by occlusion of the left coronary artery (LCA) as previously described (Zhang et al., 2015). MI rats were either injected with baicalin (MI + baicalin, 50 mg/kg/d) or saline (MI + NS, 1 ml/d) once a day for 14 consecutive days.

Abnormal Q waves or ST-segment displacements in electrocardiograms (ECGs) were observed in MI rats after surgery indicated the success of MI model.

### Sympathetic Nerve Activity

The rats were anesthetized with 10% (w/v) chloral hydrate. The left cervical sympathetic nerve and cervical artery were exposed through a left neck flank incision. The sympathetic nerve running on or beside the cervical artery was identified. The cervical sympathetic nerve was placed on a pair of platinum-iridium recording electrodes that was isolated from surrounding muscle with sterile cotton that had been soaked in paraffin oil. Neuronal electrical activity picked up by the electrodes was displayed on a computer physiograph.

### Blood Pressure and HR Measurements

Diastolic blood pressure (DBP), systolic blood pressure (SBP), and heart rate (HR) were measured by indirect tail-cuff plethysmography with a non-invasive blood pressure monitor, as previously described (Zhang et al., 2015). Systolic pulsation was detected by an electrosphygmograph coupler (ZH-HX-Z, MD 3000, Anhui, China), transduced by a pneumatic transducer and recorded on a physiograph. The rats were habituated to the test procedure during the 14 days prior to experiments, and all measurements were made by the same scientist in a quiet room. SBP was averaged over five measurements for each rat.

### Quantitative Real-Time PCR

Fourteen days post-surgery, the rats were anesthetized (35 mk/kg i.p.) and killed by decapitation. The DRGs from cervical 1 segment to upper thoracic 2 segments of both sides, which have been demonstrated to be involved in the MI, were used for real-time PCR (Liu et al., 2013). The total RNA was extracted using Trizol total RNA reagent (Beijing Tiangen Biotech, Co.). A reverse transcription reaction was completed using a RevertAid^TM^ First Strand cDNA Synthesis Kit (Fermentas, Waltham, MA, United States), according to the manufacturer’s instructions. The sequences were P2Y_12_: sense, 5′-CTTCGTTCCCTTCCACTTTG-3′, anti-sense, 5′-AGGGTGCTCTCCTTCACGTA-3′; and β-actin, sense, 5′-TGTCACCAACTGGGACGATA-3′, anti-sense: 5′-GGGGTGTTGAAGGTCTCAAA-3′. Quantitative PCR was performed using the SYBR^®^ Green Master Mix in an ABI PRISM^®^ 7500 Sequence Detection System (Applied Biosystems Inc., Foster City, CA, United States). The thermal cycling parameters were 95°C for 30 s, followed by 40 cycles of amplifications at 95°C for 5 s, and 60°C for 30 s. The amplification specificity was determined using a melting curve, and results were processed by software provided with an ABI7500 PCR instrument.

### Western Blotting

Western blotting was performed as described previously (Zou et al., 2016). Briefly, cervical dorsal root ganglions (cDRGs) and stellate ganglion (SG) of both sides were dissected 14 days after surgery, and homogenized by mechanical disruption to obtain total protein. The equal amount (40 μg) of protein was loaded in each well and was separated by SDS-polyacrylamide gel electrophoresis using a Bio-Rad electrophoresis and blotting system. The separated proteins were transferred onto PVDF membranes. The membranes were blocked with 5% (w/v) skimmed milk for 2 h at room temperature, followed by overnight incubation with rabbit anti-P2Y_12_ (1:1000, ab18441 Abcam, United Kingdom), mouse monoclonal anti-β-actin (1:800, Beijing Zhongshan Biotech Co., China), mouse anti-glial fibrillary acidic protein (GFAP, 1:500, Millipore, United States), anti-interleukin (IL)-1β (1:500, Abcam, United Kingdom), rabbit anti-phosphorylated AKT (p-AKT) and AKT (1:1000 concentration, Alomone Labs, Jerusalem, Israel) at 4°C. The membrane was incubated with horseradish peroxidase-conjugated secondary antibody (1:2000, Beijing Zhongshan Biotech Co.) for 1 h at room temperature. During Western blotting, we incubated the β-actin and targeted protein antibody at the same membrane without strip, as there’s a clear distance between the band of targeted protein and the band of β-actin according to the indication of protein maker. The membrane was cut into two parts, incubated β-actin and targeted protein, respectively, and the different two targeted protein were probed in two different membranes. The integrated optical density (IOD) of each band was quantified using Image-Pro Plus software. The IOD of the target proteins was normalized to the intensity of the respective β-actin internal control.

### Double-Labeled Immunofluorescence

Double-labeled immunofluorescence was performed, as previously described (Liu et al., 2016). Briefly, rat cervical (C1-T2) DRGs were dissected and fixed in 4% (w/v) paraformaldehyde (PFA) for 2 h at room temperature. DRGs were then dehydrated in 30% (w/v) sucrose and 4% (w/v) PFA overnight. Then, 10-μm frozen sections were obtained using a cryostat and mounted onto glass slides. Sections were washed in PBS and incubated in blocking solution containing 3% (v/v) bovine serum albumin (BSA) in PBS with 0.3% (v/v) Triton X-100 for 30 min at room temperature. Primary antibodies against GFAP (mouse anti-GFAP, Millipore) and P2Y_12_ (rabbit anti- P2Y_12_, APR-012 Alomone Labs, Jerusalem, Israel) were diluted to a ratio of 1:100. Sections were washed again in PBS and incubated with secondary antibody [(goat anti-rabbit TRITC, Jackson ImmunoResearch Inc., West Grove, PA, United States) and goat anti-mouse FITC (Beijing Zhongshan Biotech Co.)] diluted 1:200 in PBS for 1 h at 37°C. Finally, the sections were washed once more in PBS, and results were assessed using a 20× objective fluorescence microscope (Olympus, Tokyo, Japan). Data were collected from six animals in each group. Five fields containing approximately 50 neurons were randomly selected, and data analysis from each animal was averaged.

### Measurement of [Ca^2+^]_i_ in HEK293 Cells Transfected With the P2Y_12_ Receptor

Cultured HEK293 cells were transfected with the human P2Y_12_ receptor plasmid and divided into two groups: HEK293 cells transfected with the P2Y_12_ receptor as control group and baicalin-treated HEK293 cells transfected with the P2Y_12_ receptor as baicalin treatment group. Cells were loaded with fluo-3 AM (5 μM, Molecular Probes/Invitrogen Corporation, Carlsbad, CA, United States) for 40 min at 37°C in Hank’s balanced salt solution (HBSS) containing (in mM): NaCl (140), HEPES (10), CaCl2 (2), MgCl2 (1), glucose (10), and KCl (50). Cells were rinsed with HBSS and mounted in a chamber that was placed onto an inverted microscope; HBSS was perfused continuously at a rate of 1 ml min^-1^. Intracellular calcium was measured using a xenon arc lamp, interference filters, an electronic shutter (MT 20, Germany), a 20× objective (Olympus, Japan) and digital video microfluorometry with an intensified CCD camera coupled to a microscope (Olympus, Japan). The excitation wavelengths for the fluo-3 AM (485 nm) were selected using a filter changer. The P2Y_12_-specific agonist 2-(methylthio) adenosine 5′-diphosphate trisodium salt (2Me-SADP, Sigma) was applied. In all experiments a concentration of 100 μM was used to ensure maximum activation of calcium release. Cells were reconstituted in 0.1% BSA/PB. If no response was seen within 1 min, 2Me-SADP was washed out. The fluorescence ratio *F*/*F*_0_ (*F*_0_ is the baseline), was used to describe relative changes in [Ca^2+^]_i_. The ratio of peak/baseline (*F*_max_/*F*_0_) fluorescence from three random fields was analyzed using Cell RSens software [Olympus Soft Imaging Solutions (OSIS), Germany].

When cells treated with baicalin for 24 h at doses 0.1, 0.5, 1, 5, 10, 50, or 100 μM, no cellular cytotoxic effect was observed. However, significant cytotoxic effects were observed in cells treated with >50 μM for 3 days, which showed cellular viability was significantly reduced. The concentration of baicalin we used here is 10 μM, the cells were treated with bacalin for 24 h.

### Isolation and Primary Culture of DRG Satellite Glial Cells (SGCs)

The SGCs of DRG (C1-T2) from neonatal Sprague-Dawley rats were prepared as follows. Briefly, 1- to 3-day-old neonatal rats were anesthetized, and DRGs were harvested in ice-cold Hank’s balanced salt solution (HBSS). DRGs were removed with fine dissecting forceps from the inner side of each half of the dissected vertebrae together with the dorsal and ventral roots and attached spinal nerves. After removing the attached nerves and the surrounding connective tissue, DRGs were incubated with trypsin (2.5 mg/mL; type III, Sigma), collagenase (1.0 mg/mL; type IA, Sigma), and DNase (0.1 mg/mL; type IV, sigma) at 37°C in a shaking bath for 15 min. Subsequently, 10% fetal bovine serum (FBS) was added to stop enzymatic digestion. After centrifuging (5 min, 1000 rpm), the remaining ganglia were dissociated into single cells by trituration with heat-polished Pasteur pipettes and passed through nylon mesh with a pore diameter size of 100 μm. Isolated cells were suspended in Dulbecco’s Modified Eagle Medium (DMEM) (Gibco by Life Technologies) supplemented with 10% FBS and 1% penicillin/streptomycin. These cells were seeded on uncoated 35 mm dishes at 37°C with 5% CO_2_ for up to 3 days. The media were completely changed every day. Before calcium fluorescence imaging, DRG SGCs were cultured with or without baicalin (10 μM) for 24 h. The experiments were performed at room temperature (20–30°C).

### Measurement of [Ca^2+^]_i_ in SGC Cultures

The SGCs were loaded with fluo-3 AM (5 μM, Molecular Probes/Invitrogen Corporation, Carlsbad, CA, United States) for 40 min at 37°C temperature in HBSS containing (in mM): NaCl (140), HEPES (10), CaCl_2_ (2), MgCl_2_ (1), glucose (10), and KCl (50). Cells were rinsed with HBSS and mounted in a chamber that was placed onto the inverted microscope; HBSS was perfused continuously at a rate of 1 mL/min. Intracellular calcium was measured by a xenon arc lamp, interference filters, an electronic shutter (MT 20, Germany), a 20× objective (Olympus, Japan), and digital video microfluorometry with an intensified CCD camera coupled to a microscope (Olympus, Japan). The excitation wavelengths for the fluo-3 AM (485 nm) were selected by a filter changer. The P2Y_12_-specific agonist 2-(Methylthio) adenosine 5′-diphosphate trisodium salt (2Me-SADP, Sigma) was applied directly to the cover slip bathing solution after stoppage of perfusion. In all experiments a concentration of 100 μM was used to ensure maximal activation. Cells were reconstituted in 0.1% BSA/PB. If no response was seen within 1 min, the 2Me-SADP was washed out. A minimum of 50 SGCs were analyzed for each group (Castillo et al., 2013; Feldman-Goriachnik and Hanani, 2017). The fluorescence ratio *F*/*F*_0_, where *F*_0_ is the baseline, was used to describe relative changes in [Ca^2+^]i. The ratio of peak/baseline (*F*_max_/*F*_0_) fluorescence from three random fields was analyzed using Cell RSens software [Olympus Soft Imaging Solutions (OSIS), Germany] (Castillo et al., 2013; Feldman-Goriachnik and Hanani, 2017). The 40 cells for each group were examined and three independent replicates were performed.

### Molecular Docking

Molecular docking computations were performed using AutoDock 4.2 (Morris et al., 2009; Trott and Olson, 2010). Molecular docking is a computer simulation tool that attempts to predict the binding mode of a ligand in the active site of a protein. Molecular docking studies mimic the natural interaction of a ligand with the protein. The docking technique was designed to position the ligand in different orientations and conformations within the binding site to calculate optimal binding geometries and energies. Therefore, after the docking procedure, the proper conformation of the ligand in the active site of the protein is obtained and used to calculate molecular descriptors. For each ligand, a number of configurations called poses are generated and scored (Trott and Olson, 2010). The score is calculated as either a free energy of binding, which considers solvation and entropy, the enthalpic term of the free energy of binding, or a qualitative shaped-based numerical measure. The final top-scoring poses, along with their scores and conformation energies, were input into a database for further analysis.

Protein Data Bank entry NP_073637 was used as the target protein (Biasini et al., 2014; Rao et al., 2017). Baicalin, PubChem CID 64982, was used as the ligand. Both structures were prepared using AutoDockTools (ADT) (Morris et al., 2009) and Python scripts named prepare_ligand4.py and prepare_receptor4.py, which are associated with the AutoDock4.2 program. The binding pocket position in the target protein was specified with the ADT molecular viewer. The parameters were maintained at their default values. Finally, the output files were viewed using MGL tools (Morris et al., 2009) and PyMol^1^.

### Statistical Analysis

Data were analyzed using SPSS 11.5 software. Results were shown as means ± SD. Statistical significance was determined by one-way analysis of variance (ANOVA) followed by the Fisher’s *post hoc* test for multiple comparisons. *P* < 0.05 was considered to represent a significant result.

## Results

### Downregulation by Baicalin of the Expression of the P2Y_12_ Receptor in MI Rats

**FIGURE 1:**
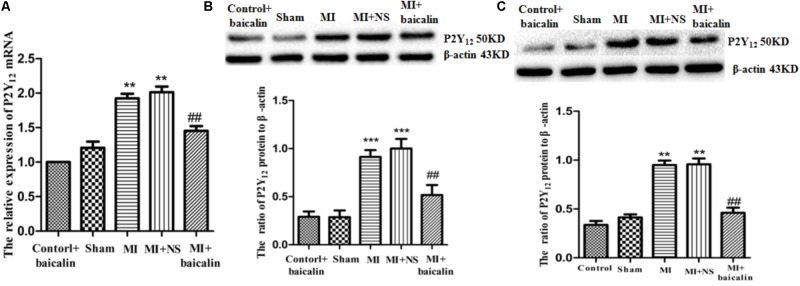
Baicalin prevented the upregulation of P2Y_12_ receptor in MI rats. **(A)** Real-time PCR assay showed that the expression levels of P2Y_12_ receptor mRNA in the DRG were higher in the MI group than in the sham group (*p* < 0.01). No statistically significant difference was found between MI and MI + NS rats. In MI rats treated with baicalin, the expression level of P2Y_12_ mRNA was lower than that in untreated MI rats (*p* < 0.01). No statistically significant difference was found between control + baicalin and sham rats. 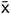 ± s, *n* = 8. ^∗∗^*p* < 0.01 compared with the sham group. ^##^*p* < 0.01 compared with the MI group. **(B)**. Western blotting assay indicated that expression levels of P2Y_12_ protein in the DRG were higher in the MI group than in the sham group (*p* < 0.001). No statistically significant difference was found between control + baicalin and sham rats. In MI rats treated with baicalin, the expression of P2Y_12_ receptor protein was lower than that in the untreated MI rats (*p* < 0.01). No statistically significant difference was found between MI and MI + NS rats. 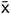 ± s, *n* = 8. ^∗∗∗^*p* < 0.01 compared with the sham group. ^##^*p* < 0.01 compared with the MI group. **(C)** Western blotting assay indicated that expression levels of P2Y_12_ protein in the SG were higher in the MI group than in the sham group (*p* < 0.01). No statistically significant difference was found between control + baicalin and sham rats. In MI rats treated with baicalin, the expression of P2Y_12_ receptor protein was lower than that in the untreated MI rats (*p* < 0.01). No statistically significant difference was found between MI and MI + NS rats. 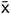 ± s, *n* = 8. ^∗∗^*p* < 0.01 compared with the sham group. ^##^*p* < 0.01 compared with the MI group.

Real-time PCR was used to detect expression levels of P2Y_12_ receptor mRNA in the DRG. The results showed that the P2Y_12_ receptor mRNA expression in the MI group was significantly higher than that in the sham group (*p* < 0.01). No difference was found between MI and MI + NS groups. After treatment with baicalin, the expression levels of P2Y_12_ receptor mRNA were decreased in comparison with the untreated MI rats (*p* < 0.01) (**Figure 1A**). No difference was found between control + baicalin and sham groups.

P2Y_12_ receptor protein expression levels in the DRG were analyzed by Western blotting. IOD values of P2Y_12_ receptor protein were normalized according to the IOD values of each β-actin internal control. Like mRNA levels, IOD values of the P2Y_12_ receptor protein levels were also significantly increased in MI group of rats compared to the sham group (*p* < 0.01). Additionally, no difference was found between MI and MI + NS groups. However, when MI rats were treated with baicalin, the expression level of the P2Y_12_ receptor protein was lower than that in untreated MI rats (*p* < 0.01) (**Figure 1B**). No difference was found between control + baicalin and sham groups.

P2Y_12_ receptor expression levels in the SG were analyzed by Western blotting. IOD values of P2Y_12_ receptor protein were normalized according to the IOD values of each β-actin internal control. IOD values of the P2Y_12_ protein levels in the SG were also significantly increased in MI group of rats compared to the sham group (*p* < 0.01). Additionally, no difference was found between MI and MI + NS groups. However, when MI rats were treated with baicalin, the expression level of the P2Y_12_ receptor protein was lower than that in untreated MI rats (*p* < 0.01) (**Figure 1C**). No difference was found between control + baicalin and sham groups. These results indicated that baicalin treatment also decreased the upregulation of the P2Y_12_ receptor in the SG to affect the pathology of MI.

### Baicalin Alleviated the Pathologic Changes in Sympathetic Nerve Activity, SBP, DBP, HR, and ECGs in the MI Rats

**FIGURE 2:**
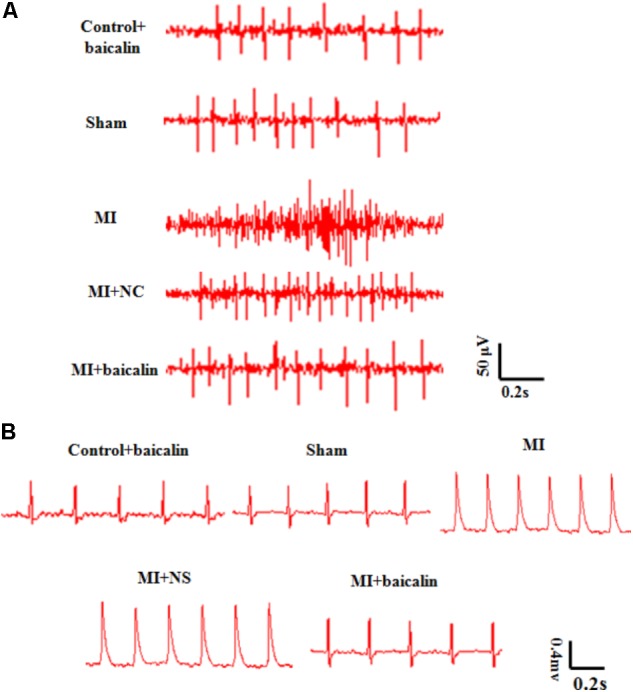
Baicalin relieved pathological changes in sympathetic nerve activity, and ECGs in MI rats. **(A)** The sympathetic nerve activity was significant enhanced after MI injury, and was reduced with baicalin treatment. **(B)** On day 14 after MI injury, there were obvious abnormal Q-waves in the MI and MI + NS groups. In MI rats treated with baicalin, Q-wave abnormalities were improved.

Sympathetic nerve activity in MI rats was significantly increased 14 days after MI, compared with that of the sham rats. The sympathetic nerve activity in MI rats treated with baicalin was alleviated in comparison with untreated MI rats. Normal saline or only baicalin treatment had no influence on sympathetic nerve activity (**Figure 2A**).

Fourteen days after surgery, SBP, DBP, and HR were significantly higher in the MI group than in the sham group. No significant differences were found between MI and MI + NS rats. SBP, DBP and HR of baicalin-treated MI rats were significantly lower than in untreated MI rats. No significant differences were found between control + baicalin and sham groups (**Table 1**).

**Table 1:** Baicalin alleviated the pathologic changes in systolic blood pressure (SBP), diastolic blood pressure (DBP) and heart rate (HR) in the myocardial ischemia (MI) rats.

Groups	SBP (mmHg)	DBP (mmHg)	HR (time/min)
Control + baicalin	108.75 ± 1.708	83.5 ± 1.291	352 ± 2.944
Sham	111.333 ± 4	85 ± 5	355.333 ± 13.868
MI	160 ± 4^∗∗∗^	101 ± 4.359^∗^	460 ± 13.229^∗∗∗^
MI + NS	155 ± 6.245^∗∗∗^	99 ± 3.601^∗^	449.67 ± 22.189^∗∗∗^
MI + Baicalin	117 ± 9.165^###^	89 ± 6.083^#^	377 ± 13.748^###^

*SBP, DBP, and HR in the MI group were significantly higher than in the sham group. No difference was found between control + baicalin and sham rats. No difference was found between the MI and the MI + NS rats. SBP, DBP, and HR in MI rats treated with baicalin were significantly reduced compared with those in the untreated MI rats. No difference was found between the control + baicalin and sham groups. 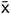 ± s, n = 8. ^∗∗∗^p < 0.001 compared with the sham group; ^∗^p < 0.05 compared with the sham group; ^###^p < 0.001 compared with the MI group, ^#^p < 0.05 compared with MI group.*

Likewise, abnormal Q-waves and ST-segment displacements were detected in the MI group when compared with the sham group 14 days post surgery. No significant differences were found between MI and MI + NS rats. Baicalin treatment improved these ECG abnormalities in MI + baicalin rats compared to MI rats that received no baicalin treatment (**Figure 2B**). No statistically significant difference was found between the control + baicalin and sham groups.

### Baicalin Decreased the Upregulated Coexpression Values of P2Y_12_ and GFAP in DRG of MI Rats

**FIGURE 3:**
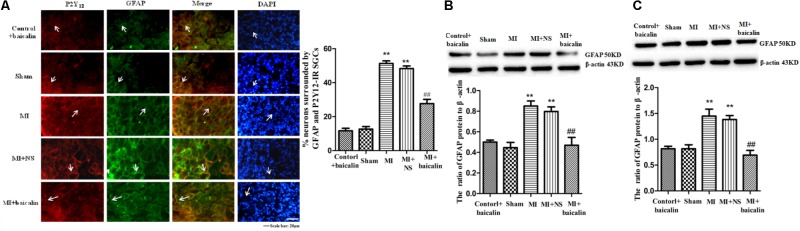
Baicalin decreased the coexpression values for P2Y_12_ receptor and GFAP in the MI rats. **(A)** Coexpression values for P2Y_12_ receptor and GFAP in the DRG SCGs were assessed by double-labeled immunofluorescence. Coexpression values for the P2Y_12_ receptor with GFAP in the MI group were higher than those in the sham group. No statistical difference was found between control + baicalin and sham rats. Coexpression values in the MI + baicalin group were lower than those in the untreated MI group. No statistical difference was found between MI and MI + NS rats. The green signal represents GFAP staining with FITC, and the red signal indicates P2Y_12_ receptor staining with TRITC. Merge represents the P2Y_12_ receptor and GFAP double-staining image. Histogram showed that the number of neurons surrounded with GFAP and P2Y_12_ positive SGCs in the SCGs. Scale bar: 20 μm. **(B)** Western blotting assay to show that the expression levels of the GFAP protein in the DRG were higher in the MI group than in the sham group (*p* < 0.01). No statistical difference was found between control + baicalin and sham rats. Baicalin treatment in MI rats appeared to decrease expression levels of GFAP in the DRG compared with the untreated MI group (*p* < 0.01). No statistical difference was found between the MI and the MI + NC rats. 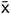 ± s, *n* = 8. ^∗∗^*p* < 0.01 compared with the control group, ^##^*p* < 0.01 compared with the MI group. **(C)** Western blotting assay to show that the expression levels of the GFAP protein in the SG were higher in the MI group than in the sham group (*p* < 0.01). No statistical difference was found between control + baicalin and sham rats. Baicalin treatment in MI rats appeared to decrease expression levels of GFAP in the SG compared with the untreated MI group (*p* < 0.01). No statistical difference was found between the MI and the MI + NC rats. 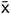
±s, *n* = 8. ^∗∗^*p* < 0.01 compared with the control group, ^##^*p* < 0.01 compared with the MI group.

Coexpression values (the number of neurons surrounded with GFAP and P2Y_12_ positive SGCs in DRG) for P2Y_12_ receptors and GFAP, a marker for satellite glial cells (SGC) in SGCs sheathing the DRG neurons were tested by double immunofluorescence. Co-expression values for P2Y_12_ and GFAP in the MI group were higher than those of the sham group. Upregulation of GFAP in DRG satellite glial cells suggests the activation of SGCs after MI injury. There was no significant difference between the MI group and the MI + NS group. Coexpression values for P2Y_12_ and GFAP were lower in MI rats treated with baicalin than in untreated MI rats. There was no significant difference between the control + baicalin and sham groups (**Figure 3A**).

The expression values for GFAP protein in the DRG were determined by Western blotting. When compared with the sham group, GFAP expression levels in the MI group were significantly increased (*p* < 0.01). No difference was found between MI and MI + NS rats. Upregulated GFAP protein levels also indicated that SGCs was activated following MI. The relative expression of GFAP was lower in MI rats treated with baicalin than in untreated MI rats (*p* < 0.01) (**Figure 3B**). There was no significant difference between the control + baicalin and sham groups. These results indicated that the upregulation of the P2Y_12_ receptor in activated SGCs was involved in the pathology of MI.

In addition, the expression values for GFAP protein in the SG were determined by Western blotting. When compared with the sham group, GFAP expression levels in the MI group were significantly increased (*p* < 0.01). No difference was found between MI and MI + NS rats. Upregulated GFAP protein levels also indicated that SGCs in the SG was activated following MI. The relative expression of GFAP was lower in MI rats treated with baicalin than in untreated MI rats (*p* < 0.01) (**Figure 3C**). There was no significant difference between the control + baicalin and sham groups. These results indicated that baicalin treatment decreased the upregulation of the P2Y_12_ receptor in activated SGCs of the SG to improve the pathology of MI.

### Baicalin Decreased the Expression Level of IL-1β in the DRG of MI Rats

**FIGURE 4:**
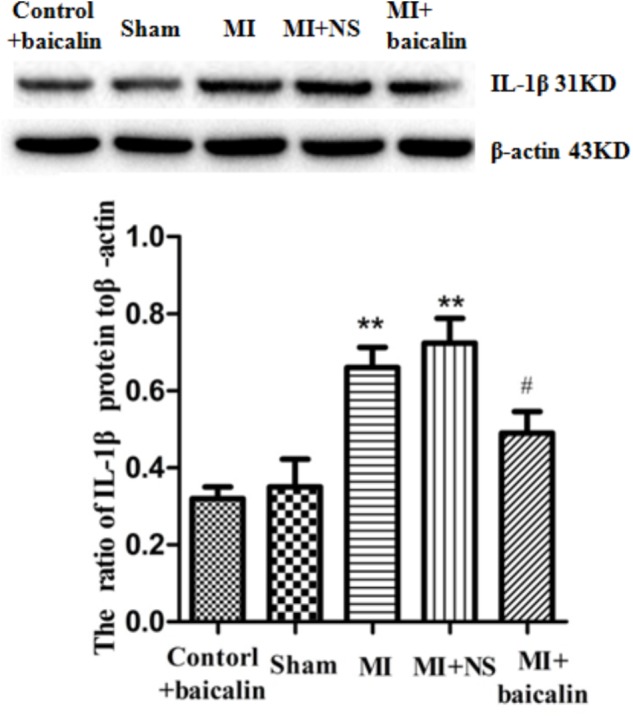
Baicalin decreased the expression levels of IL-1β in the DRG of MI rats. Western blotting assay showed that expression of IL-1β in the MI group was significantly elevated compared with the sham group (*p* < 0.01). No statistical difference was found between control + baicalin and sham rats. Relative expression of IL-1β was lower in the MI rats treated with baicalin compared with untreated MI rats (*p* < 0.05). There was no statistical difference between the MI group and the MI + NS group. 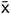
±s, *n* = 8. ^∗∗^*p* < 0.01 compared with the control group, ^#^*p* < 0.05 compared with the MI group.

The pro-inflammatory cytokine IL-1β is involved in the pathogenesis of cardiac ischemic injury. Expression levels of IL-1β protein in the DRG were determined by Western blotting. We found that the expression of IL-1β in the MI group was significantly elevated compared with the sham group (*p* < 0.01). There was no difference between the MI group and the MI + NS group. Relative expression of IL-1β was lower in MI rats treated with baicalin compared with untreated MI rats. There was no significant difference between the control + baicalin and sham groups (**Figure 4**).

### Baicalin Decreased the Activation of Akt in the DRG of MI Rats

**FIGURE 5:**
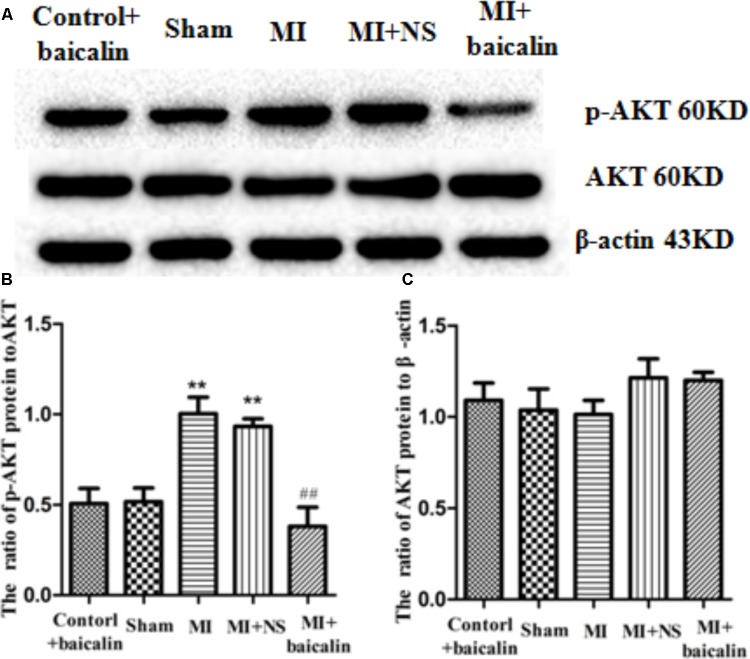
Baicalin decreased the activation of Akt in the DRG of MI rats. Western blotting assay showed that there is no statistical difference for the IOD ratio of AKT/β-actin between the MI rats and the sham rats **(A,B)**. The expression levels of p-AKT to AKT were significantly increased in the MI rats in comparison with that in the sham rats (*p* < 0.01) **(A,C)**. No statistical difference was found between control + baicalin and sham rats. In the MI rats treated with baicalin, the expression levels of p-AKT to AKT were significantly reduced compared to untreated MI rats (*p* < 0.01) **(A,C)**. No statistical difference was found between MI and MI + NS groups. 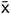 ± s, *n* = 8. ^∗∗^*p* < 0.01 compared with the control group, ^##^*p* < 0.01 compared with the MI group.

Akt phosphorylation is a marker of Akt activation. The expression levels of Akt in DRG were detected by Western blotting. The results show that there is no statistical difference in the IOD ratios of AKT/β-actin between the MI rats and the sham rats. The expression levels of p-AKT to AKT were significantly increased in MI rats in comparison with those in sham rats (*p* < 0.01). There was no difference between the MI group and the MI + NS group. Conversely, the ratio of p-AKT to AKT protein levels in MI rats treated with baicalin were significantly reduced compared with untreated MI rats (*p* < 0.01) (**Figure 5**). There was no significant difference between the control + baicalin and sham groups. These results indicated that baicalin treatment alleviated the sympathoexcitatory reflex via suppression of the phosphorylation of AKT in DRG.

### Baicalin Inhibited the P2Y_12_-Agonist-Activated [Ca^2+^]_i_ in HEK239 Cells

**FIGURE 6:**
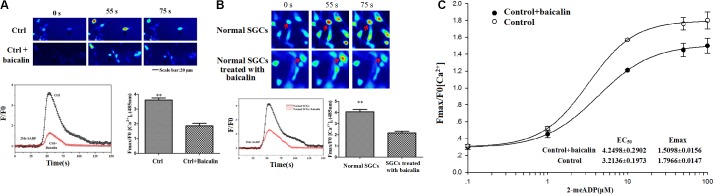
Baicalin inhibited the P2Y_12_-agonist-activated [Ca^2+^]_i_ in HEK239 cells transfected with the P2Y_12_ receptor and DRG SGCs. **(A)** Baicalin inhibited the P2Y_12_-agonist-activated [Ca^2+^]_i_ in HEK239 cells transfected with the P2Y_12_ receptor. The ratio of peak/baseline fluorescence of [Ca^2+^]_i_ activated by the P2Y_12_ agonist, 2Me-SADP, in HEK293 cells transfected with a P2Y_12_ receptor plasmid was recorded by Fluo 3-AM calcium fluorescence imaging. 24 h treatment with baicalin inhibited 2Me-SADP-mediated increases of [Ca^2+^]_i_ in HEK293 cells transfected with P2Y_12_ receptor plasmid. **(B)** Baicalin reduced the P2Y_12_-agonist-activated [Ca^2+^]_i_ in DRG SGCs. P2Y_12_-agonist-activated [Ca^2+^]_i_ in the DRG SGCs was recorded by calcium fluorescence imaging. The ratio of peak/baseline fluorescence of [Ca^2+^]_i_ activated by the P2Y_12_ agonist 2Me-SADP (100 μM) in DRG SGCs without treated with baicalin was larger than that in cells treated with baicalin group. Baicalin treatment inhibited 2Me-SADP-induced [Ca^2+^]_i_ in DRG SGCs. **(C)** The full concentration-response curve of baicalin reducing the P2Y_12_-agonist-activated [Ca^2+^]_i_ in DRG SGCs. EC_50_ and *E*_max_ of control + baicalin are 4.2498 ± 0.2902 and 1.5098 ± 0.0156, respectively. EC_50_ and *E*_max_ of control are 3.2136 ± 0.1973 and 1.7966 ± 0.0147, respectively ^∗∗^*p* < 0.01.

In Fluo-3 AM calcium fluorescence imaging, the ratio of peak/baseline fluorescence of [Ca^2+^]_i_ activated by the P2Y_12_ agonist, 2Me-SADP (100 μM) was recorded in HEK293 cells transfected with the P2Y_12_ receptor plasmid. After 24 h treatment, baicalin inhibited 2Me-SADP-induced rise in intracellular Ca^2+^ ([Ca^2+^]_i_) in transfected HEK293 cells (**Figure 6A**). These results indicated that baicalin inhibited the activation of the P2Y_12_ receptor in HEK293 cells transfected with the P2Y_12_ receptor plasmid.

P2Y_12_-agonist-activated [Ca^2+^]_i_ in DRG SGCs was measured by Fluo-3 AM calcium fluorescence imaging. The ratio of peak/baseline fluorescence of [Ca^2+^]_i_ activated by the P2Y_12_ agonist 2Me-SADP (100 μM) in DRG SGCs without treated with baicalin was larger than that in cells treated with baicalin group. Baicalin treatment also inhibited 2Me-SADP-induced [Ca^2+^]_i_ in DRG SGCs (**Figure 6B**). The full concentration-response curve was showed in **Figure 6C**. These results indicated that baicalin inhibited the activation of the P2Y_12_ receptor in DRG SGCs.

### Molecular Docking of Baicalin on P2Y_12_ Receptor

**FIGURE 7:**
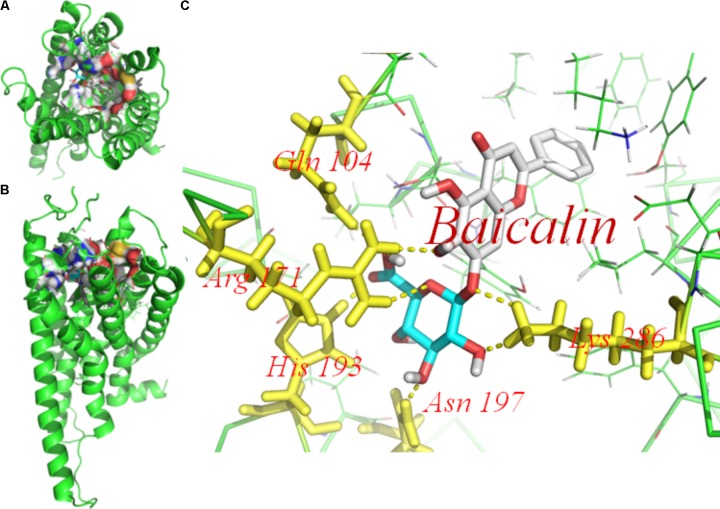
Molecular docking of baicalin on the human P2Y_12_ protein. Simulated model of baicalin docked with the human P2Y_12_ (hP2Y_12_) protein generated using a computer. Structures in **(A)** (forward map) and **(B)** (top view) show that the best docking position between baicalin and hP2Y_12_. The docking position was in the outside of the cell membrane and the entrance of the channel. **(C)** Was the docking pocket. **(C)** Showed that the yellow dotted line was a hydrogen bond between the residues on the chains of P2Y_12_ protein and baicalin. The photo indicated that there was strong binding energy between baicalin and GLN101, ARG 171, and HIS 193 in left chain, LYS 286 in right chain. The results revealed that baicalin could interact with the hP2Y_12_ protein.

Molecular docking of baicalin on a hP2Y_12_ protein was performed by AutoDock 4.2. The docking score of the hP2Y_12_ protein and baicalin (-9.3 Kcal/mol) showed that baicalin had the perfect fit to interact with the hP2Y_12_ receptor (**Table 2**). The perfect match enabled baicalin to interact with residues in the P2Y_12_ receptor agonist-binding pocket (**Figure 7**).

**Table 2:** MOE score of h P2Y_12_ protein and baicalin (kcal/mol).

Mode/rank	Affinity (kcal/mol)	Dist from best mode rmsb^∗^ l.b	Dist from best mode rmse u.b
1	-9.3	0	0
2	-8.4	3.45	7.14
3	-8.3	3.396	7.219
4	-8.2	4.353	8.495
5	-8	3.572	7.3
6	-7.5	3.757	8.106
7	-7.2	1.483	2.651
8	-7.1	3.645	5.549
9	-7	4.868	8.51

*The predicted binding affinity is in kcal/mol (Energy). ^∗^rmsd: RMSD values are calculated relative to the best mode and use only movable heavy atoms. Two variants of RMSD metrics are provided, rmsd/lb (RMSD lower bound) and rmsd/ub (RMSD upper bound), differing in how the atoms are matched in the distance calculation*.

### Comparison of the P2Y_12_ Receptor Expression in C1-T2 With Lumbar DRG1-4

**FIGURE 8:**
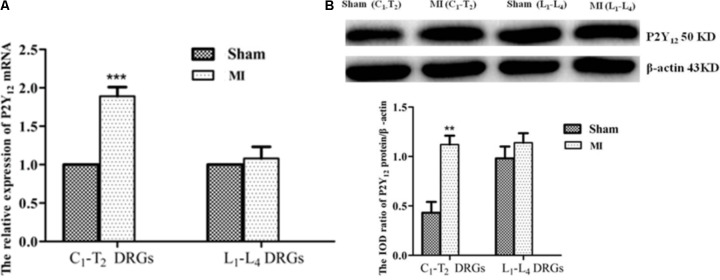
Comparison of the P2Y_12_ receptor expression in C1-T2 with lumbar DRG1-4. **(A)** Real-time PCR assay showed that the expression levels of P2Y_12_ receptor mRNA increased only in C1-T2 DRGs, with no changes in lumbar DRGs following MI. 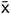 ± s, *n* = 5. ^∗∗∗^*p* < 0.001 compared with the sham group. **(B)** Western blotting assay showed that the expression levels of the P2Y_12_ protein were significantly increased in C1-T2 DRGs, with no change in the lumbar DRGs post-MI. 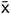 ± s, *n* = 5. ^∗∗^*p* < 0.01 compared with the sham group.

Real-time PCR and Western blot approaches were used to detect expression levels of P2Y_12_ receptor mRNA and protein. The results showed that no significant changes in the expression of the P2Y_12_ receptor mRNA and protein were observed in the lumbar DRG from the same post-MI animals (**Figure 8**), suggesting that these changes are specifically related to DRGs innervating heart rather than systemic effects associated with MI.

## Discussion

Purinergic signaling has pivotal roles in physiological and pathophysiological cardiovascular processes (Vassort, 2001; Erlinge and Burnstock, 2008; Kennedy et al., 2013; Burnstock and Pelleg, 2015). Purinergic P2Y receptors have been implicated in the treatment of cardiovascular diseases (Nishimura et al., 2017; Zou et al., 2017). In the present study, we demonstrated that both the expression levels of P2Y_12_ receptor mRNA and protein in DRG of MI rats were increased. The elevated levels of P2Y_12_ receptor mRNA and protein in DRG were decreased by baicalin treatment in MI rats. The extracellular ATP likely acts specifically as a nociceptive stimulus because epicardial application of ATP stimulates ischemia-sensitive, but not ischemia-insensitive, cardiac sympathetic afferents (Fu and Longhurst, 2010). DRGs are involved in the pathological changes and the sympathoexcitatory reflex induced by MI (Li et al., 2011; Tu et al., 2013; Liu et al., 2014). The results reported here suggest that baicalin treatment in MI rats inhibits the upregulated P2Y_12_ receptor in the DRG, which is related with ischemia-sensitive afferents.

Using horseradish peroxidase (HRP), our previous works tested by retrograde tracing showed that the cervical and upper thoracic dorsal root ganglia were related to the signaling transmission induced by MI (Liu et al., 2013; Tu et al., 2013). It was reported that changes in gene expression localized to thoracic spinal cord only, with no change in gene expression in lumber DRG after MI or infarction and the expression of immune modulators and apootic genes were observed in the thoracic DRG but not in the lumbar DRG following MI (Gao et al., 2017). Our results showed that the expression changes of P2Y_12_ mRNA and protein localized to cervical and upper thoracic spinal cord DRGs only and no changes were observed for the expression of P2Y_12_ mRNA and protein in lumbar DRGs from the same post-MI animals. Thus, the expression changes of the P2Y_12_ receptor are specifically related to DRGs innervating heart rather than systemic effects associated with MI. During a MI the cardiac sympathetic afferent nerve is activated to increase the sympathoexcitatory reflex, which includes an increase in blood pressure and HR (Armour, 1999; Wang et al., 2009; Wan et al., 2010; Li et al., 2011).

The data presented in the present study show that the upregulated P2Y_12_ receptor mRNA and protein levels in DRG were associated with the upregulation of sympathetic nerve activity. The neurophysiological studies show that endogenously produced ATP activates ischemia-sensitive, but not ischemia-insensitive, cardiac spinal afferents through both P2X and P2Y receptor mechanisms (Fu and Longhurst, 2010; Kong et al., 2013; Liu et al., 2014). The elevated sympathetic nerve activity after MI contributes toward exaggerating sympathoexcitation. SBP and HR in MI rats were both higher than in sham rats. After baicalin treatment in MI rats, SBP, DBP and HR were significantly reduced compared to the values of untreated MI rats. Additionally, pathologic changes in ECGs were improved compared to those of untreated MI rats. Hence, baicalin treatment in MI rats is able to reduce the sympathoexcitatory response. The SG as efferent nerves can directly affect cardiac sympathetic activity. Our results indicated that baicalin treatment also decreased the upregulation of the P2Y_12_ receptor, GFAP protein in the SG. Thus, baicalin treatment also reduced the cardiac sympathetic efferent activity.

The P2Y_12_ receptor is expressed in satellite glial cells (SGCs) of DRG (Katagiri et al., 2012; Kobayashi et al., 2013). SGCs envelop the cell bodies of neurons in the DRG (Hanani, 2005; Costa and Moreira, 2015). In this study, we found that the coexpression of P2Y_12_ receptor and GFAP in MI rats was higher than in the sham rats. Additionally, the expression levels of the GFAP protein in the DRG were also increased in the MI condition. Upregulation of GFAP is considered to be a marker of SGC activation. Augmented sensitivity of SGCs to ATP after MI injury may contribute to sympathoexcitatory reflex. Therefore, the upregulated P2Y_12_ receptor co-localized with GFAP in DRG SGCs in MI rats in cardiac sympathetic afferents participates in sympathoexcitatory responses after MI. Baicalin treatment in MI rats decreased co-localization values of P2Y_12_ receptor and GFAP and reduced expression levels of the GFAP protein. Hence, baicalin treatment may decrease the activation of SGCs in the DRG and inhibit the sympathoexcitatory reflex.

Satellite glial cells activation induces an increased release of pro-inflammatory cytokines, which increases neuronal excitability. Our current data demonstrates that upregulated expression of the P2Y_12_ receptor was accompanied by an increase in IL-1β levels. IL-1β release requires P2Y_12_ receptor activation via ATP (Horváth et al., 2014). Activated P2Y_12_ receptor on adjacent SGCs increases the release of the pro-inflammatory cytokine IL-1β. By releasing cytokines, SGCs increase, in turn, neuronal excitability (Takeda et al., 2007, 2009). Previous work in our laboratory has indicated that baicalin decreases P2X_3_ receptor signaling in neurons, and thereby reducing sympathetic activity (Zhang et al., 2015). The activation of SGCs is involved in the sympathoexcitatory reflex mediated by the upregulated P2X_7_ receptor via sensory-sympathetic coupling between cervical DRG nerves and cervical sympathetic ganglia nerves (Kong et al., 2013; Liu et al., 2013; Tu et al., 2013). The ability of neurons to communicate with glial cells through ATP-mediated calcium signaling has been demonstrated in DRG neurons (Verderio and Matteoli, 2011). The present results indicated that baicalin treatment of MI rats inhibited P2Y_12_ receptor activation in the SGCs, which may in turn reduced the communication between neurons and SGCs and lowered pathologically increased neuronal excitability mediated by P2X_3_ receptor activation (Zhang et al., 2015). Baicalin has anti-inflammatory and strong anti-oxidative properties (Krakauer et al., 2001). Baicalin may decrease the effects of pro-inflammatory cytokines and oxidative and reduce the sympathetic abnormal excitability.

Activation of the protein kinase B/Akt signaling pathway is involved in the transmission of nociceptive signaling (Sun et al., 2006; Xu et al., 2007). The P2Y_12_ receptor was shown to participate in Akt activation (Irino et al., 2008). The present results showed that the ratio of expression levels of p-AKT was significantly increased in MI rats in comparison with those in sham rats. After baicalin treatment, the expression levels of p-AKT were significantly reduced compared with untreated MI rats. The results further indicated that baicalin treatment decreased upregulation of the P2Y_12_ receptor, and followed by a decrease of p-Akt in the DRG of MI rats. The reduction of p-AKT in the MI rats was subsequently also correlated with the downregulation of sympathetic nerve activity. These results suggest that baicalin treatment may alleviate the sympathoexcitatory reflex of MI rats via inhibition of the protein kinase B/Akt signaling pathway in DRG.

Activation of the P2Y_12_ receptor contributes to cytosolic Ca^2+^ elevation (Pinheiro et al., 2013). HEK293 cells that a used as expression models and are known not to express endogenous receptors (Stanchev et al., 2009), were transfected with P2Y_12_ receptor plasmid were used to observe the [Ca^2+^]_i_. 2MeSADP, a P2Y_12_ receptor agonist increased cytosolic Ca^2+^ levels. The ratio of peak/baseline fluorescence of [Ca^2+^]_i_ induced by the P2Y_12_ agonist was inhibited when the transfected HEK293 cells were treated with baicalin for 24 h. Like the effect on transfected HEK293 cells, the application of baicalin also inhibited 2Me-SADP-induced [Ca^2+^]_i_ in DRG SGCs (**Figure 6B**). These results further support that baicalin treatment may inhibit the activation of the P2Y_12_ receptor. ATP released from glial cells elicits intercellular Ca^2+^ waves, exciting nearby neurons (Verderio and Matteoli, 2011). Both ATP and its metabolite ADP activate the P2Y_12_ receptor in DRG SGCs and mediate neuron-to-glia communication (Ceruti et al., 2008; Verderio and Matteoli, 2011; Magni and Ceruti, 2013). The photo in **Figure 7** indicated that baicalin interacted with hP2Y_12_. In **Table 2**, a higher value for the negative interaction energy indicates a more efficient interaction between the hP2Y_12_ receptor and baicalin. Baicalin may bind to the P2Y_12_ protein to limit the interaction between the P2Y_12_ receptor and its agonist, leading to the inhibition of the P2Y_12_ receptor. Our results suggest that inhibition by baicalin of the P2Y_12_ receptor in the DRG SGCs of MI rats was accompanied by a decrease in the communication between SGCs and neurons, followed by an inhibition of cardiac sympathetic afferent signaling. With respect to the therapeutic implications for baicalin and MI treatment and further work for the mechanism of baicalin-induced effects needs to be done to substantiate the importance of the presented data.

## Conclusion

Our results indicate that the expression levels of P2Y_12_ receptor mRNA and protein in the DRG, as well as the co-localization values of P2Y_12_ receptor and GFAP in the DRG SGCs were increased after MI. Activated SGCs increased IL-1β protein expression and elevated Akt phosphorylation. Baicalin treatment inhibited the upregulation of the P2Y_12_ receptor, GFAP protein and Akt phosphorylation in the DRG. The P2Y_12_ agonist, 2Me-SADP, induced an increase in cytosolic [Ca^2+^]_i_ in HEK293 cells transfected with the P2Y_12_ receptor plasmid and this effect was inhibited by baicalin. The pathological changes of myocardial infarction was occurring in the cervical DRGs. Baicalin may alleviate pathologic sympathetic activity induced by MI through inhibiting the afferents in the cDRG.

## Author Contributions

SDL designed research. LFZ, XYH, SML, YG, BW, ZHY, HL, SZ, TYJ, LL, LRS, CPZ, YXG, GLL, and HX performed the research. LFZ, XYH, and SML analyzed the data. LFZ and SDL wrote the paper. SDL revised the paper. All the authors read and approved the final manuscript.

## Conflict of Interest Statement

The authors declare that the research was conducted in the absence of any commercial or financial relationships that could be construed as a potential conflict of interest.

## Footnotes

1 http://www.pymol.org/
